# Valproic Acid and Breast Cancer: State of the Art in 2021

**DOI:** 10.3390/cancers13143409

**Published:** 2021-07-07

**Authors:** Anna Wawruszak, Marta Halasa, Estera Okon, Wirginia Kukula-Koch, Andrzej Stepulak

**Affiliations:** 1 Department of Biochemistry and Molecular Biology, Medical University of Lublin, 20-093 Lublin, Poland; marta.halasa@umlub.pl (M.H.); estera.okon@umlub.pl (E.O.); andrzej.stepulak@umlub.pl (A.S.); 2 Department of Pharmacognosy, Medical University of Lublin, 20-093 Lublin, Poland; wirginia.kukulakoch@umlub.pl

**Keywords:** breast cancer, valproic acid (VPA), histone deacetylase inhibitor (HDI), histone acetylation, histone deacetylases (HDACs), epigenetics, targeted therapy

## Abstract

**Simple Summary:**

Breast cancer (BC) is the most common cancer diagnosed among women worldwide. Despite numerous studies, the pathogenesis of BC is still poorly understood, and effective therapy of this disease remains a challenge for medicine. This article provides the current state of knowledge of the impact of valproic acid (VPA) on different histological subtypes of BC, used in monotherapy or in combination with other active agents in experimental studies in vitro and in vivo. The comprehensive review highlights the progress that has been made on this topic recently.

**Abstract:**

Valproic acid (2-propylpentanoic acid, VPA) is a short-chain fatty acid, a member of the group of histone deacetylase inhibitors (HDIs). VPA has been successfully used in the treatment of epilepsy, bipolar disorders, and schizophrenia for over 50 years. Numerous in vitro and in vivo pre-clinical studies suggest that this well-known anticonvulsant drug significantly inhibits cancer cell proliferation by modulating multiple signaling pathways. Breast cancer (BC) is the most common malignancy affecting women worldwide. Despite significant progress in the treatment of BC, serious adverse effects, high toxicity to normal cells, and the occurrence of multi-drug resistance (MDR) still limit the effective therapy of BC patients. Thus, new agents which improve the effectiveness of currently used methods, decrease the emergence of MDR, and increase disease-free survival are highly needed. This review focuses on in vitro and in vivo experimental data on VPA, applied individually or in combination with other anti-cancer agents, in the treatment of different histological subtypes of BC.

## 1. Introduction

Regardless of socioeconomic status and level of development of societies, cancer is one of the most common causes of morbidity and mortality worldwide [1,2]. According to the GLOBOCAN, one in six women and one in five men were diagnosed with cancer in 2018 [1]. Unfortunately, the rates of cancer incidence and mortality are still rising. It is estimated that 13 million people will die from cancer in 2030 [3].

Breast cancer (BC) is the most common cancer diagnosed among women in all regions in the world except in the eastern areas of Africa where cervical cancer occurs most often. Over the last twenty years, there has been an approximate 30% increase in the incidence rate of this disease [4]. Moreover, BC is the leading cause of neoplasms death in over 100 countries all over the world. In 2018, over 2 million new BC cases were diagnosed and nearly 630,000 deaths from BC have been reported worldwide [1,4].

Understanding the biological landscape of BC and its phenotypic heterogeneity is a key element in developing novel targeted therapies [5]. The integration of nucleic acid and peptide sequencing based on mass spectrometry and advanced biomolecular analysis allowing to define the post-translational modifications, provide a better understanding of the pathophysiology of BC, and help to develop new more effective therapeutic strategies in the treatment of this disease [6]. Unfortunately, despite numerous studies, the pathogenesis of BC is still unknown, and effective therapy of this disease is one of the most important challenges of medicine.

Both genetic and epigenetic modifications are responsible for the progression of BC. Unlike irreversible genetic alterations, epigenetic modifications can be reversible. This suggests that epigenetic changes are favored in therapeutic applications. DNA methyltransferases and histone deacetylases are the main targets for epigenetic therapy. Several inhibitors of DNA methyltransferases and histone deacetylases have been approved by the US Food and Drug Administration (FDA) as anti-cancer drugs [7,8]. Reversible histone acetylation, catalyzed by histone acetyltransferases (HATs) and histone deacetylases (HDACs), plays an important role in epigenetic regulation of gene expression. An imbalance between HAT and HDAC expression leads to the development of numerous cancers [8,9]. In most cancer cell lines, a reduction in histone acetylation levels was observed due to overexpression of HDACs activity [10]. Histone deacetylase inhibitors (HDIs) are promising new generation cytostatics that increase histone acetylation. HDIs modulate the structure of chromatin, leading to changes in the expression of genes involved in numerous signaling pathways, including induction of apoptosis, cell cycle arrest, and inhibition of angiogenesis [11]. However, the mechanism of antitumor activity and the specificity of HDIs have not been fully understood.

In our review article, we described the current state of knowledge of the use of valproic acid (VPA), short-chain fatty acid, representative of the HDIs which has been successfully used in the treatment of epilepsy, bipolar disorders, and schizophrenia for over 50 years, individually or in combination with other active agents, in the treatment of BC, with particular emphasis on the progress that have been done in this topic recently.

## 2. Molecular Subtypes of Breast Cancers and Limitations in the Therapy of Patients Harboring These Subtypes

BC is a complex group of diseases with specific pathological features and clinical implications. Extensive evidence suggests that BCs with varied biological and histopathological characteristics develop differently, resulting in miscellaneous responses to the treatment, and therefore various therapeutic strategies should be used [12,13,14].

Classic immunohistochemistry markers such as expression of estrogen (ER), progesterone (PG), and human epidermal growth factor (HER2) receptors; and clinicopathological factors, like tumor grade, size, nodal involvement, are conventionally used to select therapy and to predict disease progression. The widespread use of high-throughput techniques for gene expression analysis has shown that the response of cancer cells to treatment is not due to prognostic factors of anatomical origin, but to internal molecular characteristics of BCs [12,15].

**Figure 1:**
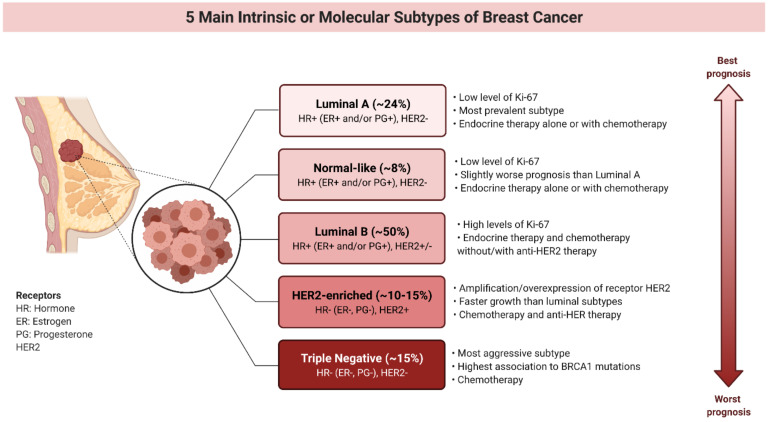
5 main intrinsic or molecular subtypes of breast cancer (BC) (ER-estrogen receptor, PG-progesterone receptor, HER2-human epidermal growth factor receptor, Ki67-proliferation index marker) [12,13,16,17].

Five molecular subtypes of BC, including luminal A, luminal B, HER2-overexpressed, triple-negative and normal-like, were identified (Figure 1) [12,15].

The hormone receptors (ER and PG) positive subtypes of BC are the most common types of breast carcinoma, among these luminal A and luminal B forms are more prevalent [18]. Luminal A BCs have a higher level of expression estrogen-related genes and lower expression of proliferative markers (Ki67 < 20%) compared to luminal B type (Ki67 ≥ 20%). Moreover, luminal B cancers are usually characterized by higher histological grade than luminal A tumors. At the molecular level, luminal A subtypes are associated with somatic mutations in *GATA3* (GATA binding protein 3), *PIK3CA* (phosphatidylinositol-4,5-bisphosphate 3-kinase catalytic subunit alpha) and *MAP3K1* (mitogen-activated protein kinase kinase 1) genes, and often exhibit *cyclin D1* overexpression. Luminal B tumors show frequent mutations in the *TP53* and *PIK3CA* genes as well as dysregulations in the retinoblastoma and MAPK (mitogen-activated protein kinase) signaling pathways [13]. Luminal cancers respond well to hormone-related therapies [12,19,20].

Unlike the luminal type, HER2-overexpressed and triple-negative breast cancer (TNBC) subtypes are characterized by a lack of expression of ER and PG receptors, as well as high aggressiveness [18]. Although HER2-overexpressed BCs carry a poorer clinical prognosis compared to luminal subtypes, they have a much better response and sensitivity to anthracycline [21] and taxane-based neoadjuvant chemotherapy [22,23]. Even though the therapy of patients expressing HER2 receptors has been revolutionized by the introduction of anti-HER2 monoclonal antibodies (e.g., trastuzumab, bevacizumab, lapatinib) [24,25,26], recurrence and development of metastasis are serious clinical issues. In addition, not all patients with HER2-overexpression respond to therapy with trastuzumab. C-X-C chemokine receptor type 4 (CXCR4) up-regulation and phosphatase and tensin homolog (PTEN) loss are associated with resistance to treatment with trastuzumab. Therefore, new therapies are being sought for the treatment of cancers resistant to anti-HER2 monoclonal antibodies [12,27].

Approximately 15% of patients suffering from BC are diagnosed with its most severe form-TNBC. TNBC characterizes lack or low expression of hormone and HER2 receptors as well as a high level of basal markers, such as keratin or epidermal growth factor receptor (EGFR). There is also increased activation of the *WNT* signaling pathway and frequent mutations in the *TP53* and *BRCA1* genes [13]. Therefore, standard hormone therapies and targeted therapy directed against HER2 are excluded. TNBC characterizes a very aggressive clinical course, and a higher risk of local and systemic relapse [12,28,29,30]. TNBC has the worst prognosis of all the BC subtypes and is treated with systemic chemotherapy to which it responds better than other subtypes. Unfortunately, the use of traditional cytostatics (cisplatin, paclitaxel) is limited by numerous side effects (bone marrow damage, severe renal failure, peripheral neuropathies), as well as the occurrence of resistance to therapies [31,32,33,34]. Due to the lack of recognized molecular targets for therapy, TNBC is an object of interest for clinical trials with novel treatment approaches [13].

Normal-like type of BC accounts 7.8% of all cancer cases and characterizes similar immunohistochemistry status to the luminal A subtype (HR+ (ER+ and/or PG+), HER, low Ki-67) and normal breast tissue profiling. The tumor necrosis factor alpha (TNFα) pathway activity increased gradually from luminal A, luminal B, normal-like, HER2-enriched and TNBC subtypes [35]. Still, while normal-like BC has relatively good prognosis, its outlook is slightly worse than luminal A cancers’ prognosis [12]. Similar to the luminal A subtype, normal-like signature was found significantly less expressed in metastatic tumors than in primary tumors. It has been demonstrated that both normal-like and luminal A signatures show a negative correlation between time to tumor recurrence (TTR) and the magnitude of gene/signature expression changes between primary and metastatic disease [36]. Interestingly, normal-like cancer is less sensitive to paclitaxel- and doxorubicin-containing preoperative chemotherapy than the TNBC and HER2+ subtypes [37].

The presence or absence of receptors characteristic of BC allows to use of specific targeted therapies and the personalized treatment of BC patients. Targeted agents acting at the epigenetic level are currently being investigated in the treatment of different hematological malignancies and solid tumors.

## 3. Histone Deacetylase Inhibitors (HDIs)

The abnormal histone acetylation profile leads to numerous cellular disorders, including tumor initiation and progression [38]. It has been shown that histone acetylation disturbances are an important factor in the progression of BC. Studies linked with abnormal acetylation level of histones in BC focus on molecular mechanisms of BC development, identification of novel biomarkers for prediction aggressiveness of the tumor, and therapeutic potential [39].

**Figure 2:**
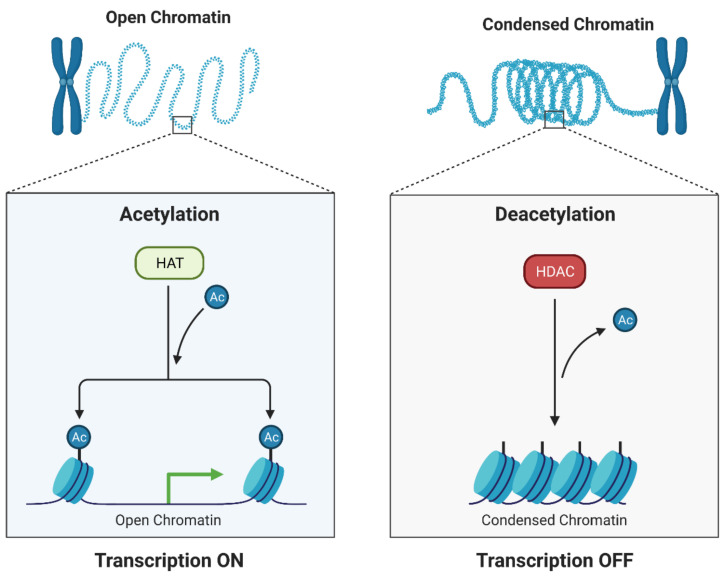
Histone acetylation modifying enzymes (HAT histone acetyltransferase, HDAD-histone deacetylase) control the transcription process by changing the status of histone acetylation and conformation of chromatin [38].

Histone acetylation modifying enzymes control the transcription process by changing the status of histone acetylation as well as other transcription factors occurring mainly in the promoter region (Figure 2) [38]. Equilibrium in the activity of the opposing enzymes: HATs and HDACs is necessary to maintain epigenetic regulation of gene expression [40]. HATs catalyze the reversible acetylation reaction at the ε-amino group of lysine residues. Neutralization of the positive charge of lysine residues due to histone acetylation is correlated with chromatin relaxation and increased transcriptional activity of genes. Unlike HATs, HDACs remove acetyl groups leading to condensation of chromatin and silencing the transcriptional activity of genes (Figure 2) [8,41].

Based on the yeast protein homology and functional criteria, HDACs were divided into four classes: zinc- (I, II and IV) and NAD-dependent (III). HDACs 1, 2, 3 and 8 belong to the I class. The II class is divided into two subclasses IIa (HDAC4, 5, 7, 9) and IIb (HDAC6, 10). Class III due to homology to silent information regulator 2 (SIR2) of *Saccharomyces cerevisiae* is called sirtuins and includes SIRT1-SIRT7. Class IV contains only one member HDAC11 (Table 1) [8,41].

Due to the fact that HDACs exert a significant effect on chromatin remodeling, their inhibitors (HDIs) have become an interesting field of study. HDIs are divided into four classes: hydroxamic acids (i.e., trichostatin A (TSA), vorinostat (SAHA), belinostat (PXD-101), panobinostat (LBH-589), resminostat (4SC-201)); short chain fatty acids (i.e. sodium butyrate (NaB), phenylbutyrate (PBA), valproic acid (VPA)); cyclic peptides (i.e. apicidin (CAS183506-66-3), romidepsin (FK228)); benzamides (i.e. mocetinostat (MGCD103), entinostat (MS-275), domatinostat (4SC-202)) (Table 2) [10].

**Figure 3:**
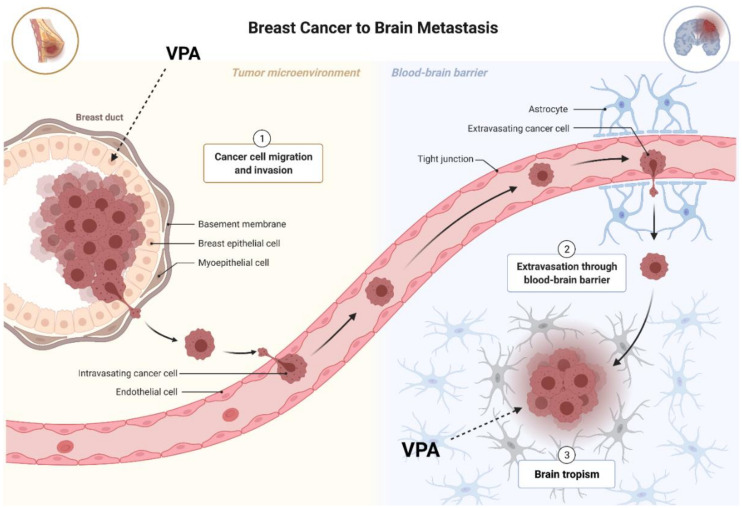
The potential effects of valproic acid (VPA) on breast cancer cells and metastatic breast cancer (BC) cells in the brain.

So far, four HDIs have been approved by the US Food and Drug Administration (FDA) for the treatment of certain types of cancer: SAHA-for the treatment of cutaneous manifestations of cutaneous T-cell lymphoma (CTCL) in patients with the progressive, persistent, or relapsing disease [42]; LBH-589-in polytherapy with bortezomib and dexamethasone for therapy of patients with relapsed and/or refractory multiple myeloma [43]; FK228 and PXD-101-for the treatment of peripheral T-cell lymphomas (PTCLs), a rare disease belonging to non-Hodgkin lymphomas [44,45]. Therefore, HDACs modulators may also be used as potential drugs in the BC treatment [39]. HDIs via inhibition of HDACs activity, increase the acetylation level of both histone and non-histone proteins [46,47] maintaining a global cellular acetylation profile which enables the activation of genes responsible for inhibiting the progression of BC. Results from pre-clinical and clinical studies have shown that HDIs can induce different anti-cancer mechanisms in many types of BC [8,40,41]. Since VPA, as a psychoneurological drug, crossing the blood-brain barrier (BBC), it could also effectively eliminate metastatic BC cells in the brain of patients (Figure 3) [48].

HDIs are able to inhibit proliferation and induce the differentiation and apoptosis of tumor cells resistant to different cytostatic drugs by regulating the expression several genes. It was already demonstrated in 2004 that administration of TSA to BC cells resistant to tamoxifen caused an increase in estrogen receptor expression, which in turn allowed for re-sensitization of these cells to the administered drug [49]. In addition, it has been shown that new synthetic HDI-FA17 overcome multidrug resistance (MDR) in BC cells of the MCF-7/MTX insensitive to methotrexate cell line [50]. In general, HDIs can induce tumor growth inhibition and apoptosis of tumor cells. Interestingly, in opposite to standard cytostatic agents, HDIs show significantly lower toxicity to normal cells [8,40,41].

## 4. Valproic Acid and Breast Cancer

**Figure 4:**
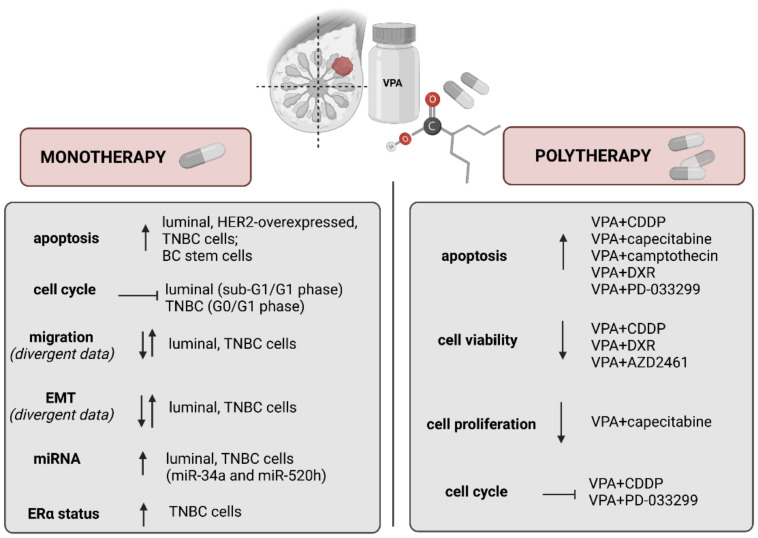
The functional mechanism of valproic acid in breast cancer treatment (BC-breast cancer, CDDP-cisplatin, DXR-doxorubicin, ER-estrogen receptor, EMT-epithelial-mesenchymal transition, HER2-human epidermal growth factor receptor 2, TNBC-triple-negative breast cancer, VPA-valproic acid) (↑—increase, ↓—decrease, 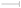
—stop).

Valproic acid (2-propylpentanoic acid, VPA) belongs to the group of short-chain fatty acids. VPA causes acetylation of the N-terminal tails on histones H3 and H4, and inhibits the activity of HDAC I and II, probably by binding to the catalytic center, and in consequence, blocking access to the substrate [8,51]. VPA has been approved by the FDA for the treatment of epilepsy and other convulsive diseases and has been used successfully in the therapy of these diseases for over five decades [52]. It has been demonstrated that VPA shows anticancer activity (Figure 4) in a diversity of human cancers [53,54,55], including breast carcinoma [56,57,58,59].

### 4.1. VPA Induces Apoptosis and Inhibits Cell Cycle

VPA decreases cell viability through arresting of the cell cycle in G1 or sub-G1 phases, induction of p21 protein expression and apoptosis by upregulation of Bak, downregulation of Bcl-2 expression, increasing Bax/Bcl-2 ratio, and, as a consequence, decreasing telomerase activity in estrogen-positive MCF7 BC cells. Telomerase is a ribonucleoprotein reverse transcriptase involved in the elongation of the telomeres and is responsible for the phenomenon of resistance to apoptosis in cancer cells [60,61]. VPA reduced proliferation not only MCF-7 BC cells but also MCF-7 BC stem cells in a time (24, 48, 72 h) and dose (0.6–20 mM) dependent manner. Cancer stem cells (CSCs) are a subpopulation of cells that reinitiate carcinogenesis, induce resistance to chemotherapy, are prone to develop metastases, and lead to disease relapse due to acquired resistance to apoptosis. Epigenetic alterations play a pivotal role in the regulation of stemness and also have been implicated in the development of drug resistance. It has been detected that MCF-7 stem cells were much more resistant to VPA than MCF-7 cells. Moreover, VPA increased levels of M30 protein (cytokeratin 18 neoepitope), caspase 3 and 7 activations, annexin-V-FITC positivity, suggesting apoptosis induction in BC stem cells. The late stage of apoptosis (secondary necrosis) was also evidenced by nuclear pyknosis with propidium iodide staining [61].

Similar to receptor-positive BCs, VPA induces cell cycle inhibition and apoptosis in BC cells with HER2 overexpression. HER2-overexpressed BC cells are more sensitive to VPA than HER2-negative ones. It has been demonstrated that the anti-proliferative mechanism of VPA in BC cells is related to their HER2-expression status. Therefore, VPA may synergize with drugs used in the therapy of HER2-overexpressed BC, like anti-HER2 monoclonal antibodies (e.g., trastuzumab, bevacizumab, lapatinib) or anthracycline and taxane-based neoadjuvant chemotherapy to inhibit HER2-overexpressing BC cell proliferation more effectively. The antiproliferative effect of VPA results from Hsp90 dysfunction which is involved in hyperacetylation of Hsp70 (non-histone protein acetylation). Hyperacetylation of Hsp70 directly affects the HER2 receptor protein, which is the client of the Hsp90 protein. The loss of Hsp90 function leads to the degradation of Hsp90 client proteins and the process of apoptosis. The alteration of the level of cyclin-dependent kinase inhibitor p21/WAF1, cleaved caspase-3, acetylated heat shock protein (Hsp) 70, acetylated Hsp90, and acetylated α-tubulin by VPA was determined in SKBR3 HER2-overexpressing BC cells. It has been observed that VPA upregulates expression and induces targeting of p21 WAF1, cleaved caspase-3, upregulates Hsp 70 acetylation, inhibits differentiation, and exhibits antiproliferative activity in BC cells in a dose- and time-dependent manner [8,62]. It has been demonstrated that VPA also remarkably inhibits the growth and triggers apoptotic cell death through G0/G1 arrest in MDA-MB-231 TNBC cells (Table 3) [57].

### 4.2. VPA Regulates Migration and Epithelial-Mesenchymal Transition (EMT)

Sodium valproate (VPA-derivative) at concentrations of 0.8–3.2 mM inhibits migration of MDA-MB-231 TNBC cells in a dose-dependent manner by upregulation of nm23H1 gene expression [65]. Nm23 (non-metastatic clone 23), also known as ndpk (nucleoside diphosphate kinase), is a metastasis suppressor gene locating on codon 21.3 of the long arm of chromosome 17. The Nm23H1 protein acts as an upstream regulator that modulates downstream metastasis-related genes, which results in tumor metastasis inhibition. Overexpression of nm23H1 gene decreases proliferation, invasion, and metastasis of cancer cells, probably mediated by nm23h1 regulation by the HDACs. It has been reported, that silencing of nm23H1 resulted in an increased in rac (Rac family small GTPase 1) gene expression and, in consequence, in the invasive ability of TNBC cells. Overexpression of nm23H1 can be a promising prognostic indicator linked with longer overall survival of patients harboring various types of cancers, including BC. However, the mechanism by which Nm23H1 participates in tumor metastasis is not fully understood [65,68,69].

Besides inhibition of cell migration, VPA affects the epithelial to mesenchymal transition (EMT). EMT is an important process of transdifferentiation in solid cancers progression and the development of metastasis. During EMT polarized, immotile epithelial cells are transformed into migratory mesenchymal-like cells prone to migration, metastasis formation, drug resistance, and BC stemness features development [70,71,72,73]. Numerous signaling pathways are involved in the EMT process, including: cadherin [74], notch [75], transforming growth factor-β (TGF-β) [76], matrix metalloproteinases [77], urokinase plasminogen activator [78] and WNT/beta-catenin [79,80] pathways [70]. However, understanding of the crosstalk of multisignaling pathways as well as assemblies of key transcription factors involved in the EMT process remains incomplete [10,70]. It has been demonstrated that VPA in concentration 1 mM does not affect cancer cell proliferation, whereas significantly increases the migration and induces EMT-like properties of MCF7 luminal and MDA-MB-231 TNBC cells via upregulation of Snail and Zeb1 transcription factors expression. Moreover, knockdown of Snail and Zeb1 attenuate VPA induced cell migration and EMT process. VPA increases the Snail protein stability through suppression of its phosphorylation at serine 11 (Ser 11). VPA also increases the transcription and promoter activity of Zeb1 via HDAC2-dependent manner. HDAC2 overexpression blocks VPA-induced Zeb1 expression [63]. In line with these findings, another research group confirmed that VPA induces cell migration and EMT process in TNBC cells through a significant increase of Snail expression and downregulation of E-cadherin and GKS3β levels. Interestingly, the levels of β-catenin and AKT were reduced after VPA treatment, suggesting that AKT/GSK3β/β-catenin signaling pathway does not mediate EMT activation [57]. Increased migration and EMT are usually associated with the worst prognosis tumors; thus, the use of VPA in monotherapy of metastatic BC may be limited. The EMT is a process characteristic of solid tumors, therefore the low therapeutic effectiveness of HDIs in this type of neoplasms may be associated with the activation of EMT process.

Changes in the expression of cadherins, so-called cadherin switches, are used very often to monitor the EMT process in development and tumor progression, in particular migration and invasion potential. It has been demonstrated that VPA inhibits the proliferation and migration in a time- and dose-dependent fashion, regardless of the BC cell type. However, BC cells with the more mesenchymal phenotype (MDA-MB-468) were found to overexpress N-cadherin, whereas BC lines with an epithelial phenotype (T47D, MCF7) responded to HDI treatment through a significant increase in E-cadherin expression. Therefore, the authors conclude that HDI induction or reversal of EMT is not a universal mechanism, yet inhibition of cell migration is, and thus EMT should not be considered as the only measurement for tumor aggressiveness [64]. Taking into account the very divergent results regarding the influence of VPA on the EMT process this phenomenon should be thoroughly re-examined using the entire panel of BC cell lines and in vivo models (Table 3).

### 4.3. VPA Affects microRNAs (miRNAs) Expression

MicroRNAs (miRNAs) are small RNAs that suppress gene expression through their interaction with 3’untranslated region in specific target mRNAs. Non-coding RNAs (ncRNAs), including miRNAs exert critical function in the regulation of cellular processes that are involved in the EMT, as a result, some miRNAs impact cancer stemness and drug resistance. Therefore, understanding the relationship between EMT and miRNAs is beneficial to both basic research and clinical treatment [81,82,83,84]. The impact of VPA on miR-34a [85], miR-520h [81], and their target gene HDAC1 expression, as well as their involvement in the induction of apoptosis in MCF-7 and MDA-MB-231 BC cell lines, were evaluated. miRNA-34a is a well-described EMT-inhibiting miRNA [85]. In the beginning, possible target genes of miR-34a and miR-520h as well as their role in apoptosis regulation were investigated using bioinformatics analyses. Potential targets of hsa-miR-34a-5p and hsa-miR-520h-5p were in silico evaluated using predictive databases, then, a functional enrichment analysis was performed with the resulting target genes, to determine genes involved in apoptosis pathway and expressed in BC tissue. Furthermore, the interactions of the potential target genes with each other as well as with hsa-miR-34a-5p and hsa-miR-520h-5p were evaluated using STRING where 56 potential interactions between the above-mentioned miRNAs and apoptosis genes were described. It has been demonstrated that VPA increases the expression of miR-34a and miR-520h and decreases HDAC1 expression in MCF-7 cells. In turn, VPA decreased the expression of these miRNAs and increased the HDAC1 expression in MDA-MB-231 BC cells. Similarly like in MDA-MB-231 BC cells, in cancer tissue the expression of miR-34a and miR-520h significantly decreased, while the expression of HDAC1 increased after VPA treatment in vivo. This raises the possibility that VPA differently regulates the expression of the same genes, depending on BC cancer type or their molecular profiling (Table 3) [56].

Changes in the miRNA level were observed after VPA treatment also in pancreatic [86], colon [87] or thymic carcinomas [88], as well as acute myeloid leukemia [89]. VPA treatment induced expression of ErbB family members-targeting microRNAs (miR-133a, miR-133b, miR-125a, miR-125b, and miR-205) in pancreatic cancer cells without altering mRNAs levels of EGFR, ErbB2, and ErbB3 [86]. A role for these miRNAs has been demonstrated in BC. miR-133 targets YES1 proto-oncogene and inhibits the growth of TNBC cells [90]. MiR-205 suppresses the malignant behaviors of BC cells by targeting CLDN11 via modulation of the EMT [91]. It has been also demonstrated that the expression of the tumor-suppressing microRNA-125b decreased in samples of BC expressing HER2 and ER, and in TNBC [92]. In turn, treating colon cancer cells with VPA reduces the levels of precursor-miR17-92a and mature miR-92a, as well as c-Myc [87]. The upregulation of miR-92a-3p was detected in tamoxifen-resistant BC cells, suggesting that lower level of miR-92a-3p could effectively improve the therapy with this drug [93]. It was also found that VPA treatment of TC1889 cells (thymic carcinoma) led to miR-145-5p up-regulation and concomitant down-regulation of miR-145-5p target genes and exhibited antitumor effects, including cell cycle arrest and the reduction of cell viability, migration capability and, colony-forming ability [88]. miR-145-5p suppresses BC progression by inhibition of (sex-determining region Y)-box 2 (SOX2) [94], a transcription factor that is essential for maintaining self-renewal and pluripotency in BC cells [95]. VPA treatment downregulated expression of CHEK1, RAD51 as well as TYMS genes, which were identified as putative targets of miR-15a and miR-16 in acute myeloid leukemia [89]. It was demonstrated that miR-15a/miR-16 induces mitochondrial-dependent apoptosis in BC cells by suppressing oncogene BMI1 [96]. The expression and role of individual mRNAs depend on the type of tumor; thus the potential effect of VPA on individual miRNAs requires further research.

### 4.4. VPA and Estrogen Receptor Status

It has been demonstrated that VPA affects not only cell migration, proliferation, and cancer cell survival, but also the expression and activation of hormone receptors in BC cells observed in the pre-clinical and clinical studies [97]. Therefore, VPA can be valuable drugs in BC therapy where ER receptor is silenced by epigenetic modifications. It has been reported that ER receptor can be indirectly activated by sub-therapeutic doses of VPA. Sub-therapeutic concentrations of VPA (100 µM) can mimic estrogen and induce growth in an estrogen-depleted medium. This effect was abolished by adding an estrogen receptor antagonist. Nonetheless, therapeutic doses of VPA act via mechanisms unrelated to the stimulation of estrogen [98]. Interestingly, MDA-MB-231 ER-negative BC cells re-activate ER receptor expression and function after VPA treatment. It has been reported that VPA induced mRNA and protein expression of ER-alpha, while did not modify the level of ER-beta. Consequently, VPA increased expression of the ER-related transcription factor FoxA1, induced inhibitory effect of tamoxifen on cell growth, and caused estradiol-induced up-regulation of estrogen-regulated genes (e.g., pS2, progesterone receptor). Summarizing, VPA inducing ER-alpha and FoxA1, conferred to MDA-MB 231 cells an estrogen-sensitive phenotype, restoring their sensitivity to anti-estrogen therapy (Table 3) [66].

### 4.5. VPA Affects Metabolic Pathways

Metabolomics is a post-genomic research area comprising different analytical methods for small molecules analysis [99]. Metabolomics is a promising strategy to explain the pathogenesis of cancer and identify new targets for cancer diagnosis and therapy [67]. The effect of VPA on metabolites and metabolic pathways in BC cells was determined. Metabolomic analysis based on UPLC-MS/MS allowed for the identification of 3137 differential metabolites of VPA in MCF-7 cells and 2472 metabolites in MDA-MB-231 BC cells after VPA treatment. VPA particularly affected the beta-alanine, taurine, and hypotaurine metabolism pathways. Expression of furfural was up-regulated after VPA treatment in both BC cell lines. All these findings can contribute to the identification of new targets for BC treatment (Table 3) [67].

## 5. “Valproic Acid et al.” and Breast Cancer

Despite the significant progress in the development of novel therapeutic options, standard chemotherapy of cancers still does not bring satisfactory results [58,100,101]. Chemotherapy with the use of standard cytostatics or their derivatives is limited due to many adverse effects, high toxicity to normal cells, or the occurrence of chemotolerance [102]. Therefore, combinations of established anticancer chemotherapeutics and new active agents with different mechanisms of action are being tested to increase the effectiveness of the therapy and improve clinical outcomes of oncological patients [58,103]. New active compounds approved for the treatment of BC, which effectively and selectively eliminate BC cells, and additionally enhance anticancer properties of currently used chemotherapeutics without destroying healthy tissue, are of great importance [11,59]. It has been demonstrated that new active agents not only reduce the effective doses of chemotherapeutic drugs but also sensitize cancer cells to standard cytostatics, increase the combined therapeutic activity of both active compounds and limit cytotoxic effect in relation to human normal cells through lowering the therapeutic doses of standard cytostatics by partially replacing them with new less toxic active agents. Additionally, combined therapy with new active agents can diminish multidrug-resistance (MDR) of currently used chemotherapeutic regimens [103]. In this context, many natural [103,104,105,106] and synthetic chemical compounds, including HDIs [11,58,59], have been identified and have become an interesting class of active agents for combined anti-cancer therapy.

Cisplatin (CDDP) or cis-diamminedichloroplatinum (II) is a well-known chemotherapeutic drug widely used in the therapy of numerous human cancers including lung, head and neck, bladder, ovarian, testicular, or TNBC. The mechanism of action of CDDP is linked to its ability to crosslink with the purine bases on the DNA, interfering with DNA repair mechanisms, causing DNA damage, and subsequently inducing apoptosis pathways in cancer cells. However, due to drug resistance and numerous adverse effects such as kidney insufficiency, gastrointestinal disorders, allergic reactions, hemorrhage, decreased immunity to infections, and hearing loss, the use of CDDP is limited [107]. The combination of VPA and CDDP resulted in the induction of apoptosis and cell cycle arrest in G1 phase in receptor-positive as well as TNBC cell lines in comparison to CDDP-monotherapy. CDDP with VPA applied together at a fixed-ratio of 1:1 exerted additive or additive with the tendency towards synergy interactions in the viability of MCF7 and T47D cells, respectively. In contrast, antagonistic (sub-additive) interaction was observed for the combination of CDDP with VPA in MDA-MB-231 TNBC cell line [59]. Interestingly, combined treatment of VPA and CDDP in MDA-MB-231 BC cells with the altered (increased or decreased) activity of Notch1 receptor yielded additive interaction. Therefore, VPA might be considered as potential therapeutic agents in combination therapy with CDDP against TNBC with altered Notch1 activity (Table 4) [58].

5-fluorouracil (5-FU) is one of the oldest chemotherapeutic drugs routinely used in single or combined modalities with other chemotherapeutic agents in the therapy of a variety of solid tumors, including BC. Mechanism of action of 5-FU has been attributed to the production of cytotoxic metabolites that are incorporated into DNA and RNA, and inhibiting thymidylate synthase, finally leading to apoptosis and cell cycle arrest in cancer cells [114]. Resistance to 5-FU is a serious clinical problem in cancer therapy, and overcoming it is a challenge for chemotherapy. HDIs can overcome resistance to various anti-cancer drugs in vitro. It has been reported that VPA increases the sensitivity of MDA-MB-468 TNBC cells to 5-FU in 5-FU sensitive and 5-FU resistant BC cells (Table 4) [108].

Capecitabine is an oral prodrug of FU, which is approved for the treatment of metastatic BC in different settings [115]. The combined therapy of VPA and capecitabine resulted in synergistic or additive antiproliferative and pro-apoptotic effects in BC cells in vitro and in vivo. It has been demonstrated that low anticonvulsant dosage of VPA induces the time- and dose-dependent up-regulation of thymidine phosphorylase (TP) gene and its protein expression in BC cells, however, TP level remains unchanged in the non-tumorigenic MCF-10A cells. TP is a key enzyme requires for its conversion of FU to 5-FU in tumors. HDAC3 was the main isoform whose inhibition was involved in the modulation of TP activity. Thereby, the combination of VPA and capecitabine could be regarded as an innovative anti-cancer strategy for the therapy of BC (Table 4) [8,109].

Camptothecin is a naturally occurring alkaloid derived from the Camptotheca acuminate. Camptothecin forms a stable ternary complex, prevents normal DNA re-ligation, and causes the complex to collide with the replication fork, leading to a DNA double-strand break [116,117]. It has also been demonstrated that VPA and camptothecin applied together induce caspase-dependent apoptosis through modulation of anti- and pro-apoptotic gene expression and loss of the mitochondrial membrane potential in MCF7 BC cells, whereas neither compound alone could efficiently induce apoptosis. It has been demonstrated that Bcl-xL expression was induced in MCF-7 BC cells treated with camptothecin alone, in contrast to cells treated with camptothecin and VPA together. Induction of apoptosis was completely suppressed by the ectopic of Bcl-xL overexpression in MCF-7 cells. Camptothecin and Bcl-xL inactivation with using siRNA or BH3 mimetic caused efficient induction of apoptosis in these cells. The cytotoxic effect of camptothecin in combination with VPA was more than additive in MCF-7 BC cells, therefore simultaneous administration of VPA and camptothecin can be a useful strategy for therapy of BC (Table 4) [110].

Doxorubicin (DXR) is a member of the anthracycline family and is currently the most effective chemotherapeutic drug used in the treatment of early and advanced breast cancer. Unfortunately, it has been demonstrated that DXR can induce drug resistance which limits the effectiveness of the agent in single-drug treatment regimes. However, the exact mechanisms of drug resistance are still poorly understood [118,119]. Sodium valproate significantly enhanced the cytotoxicity of DXR and stimulated apoptosis induced by DXR in vitro. Exposure to sodium valproate and DXR in combination resulted in significantly increased early and late cell apoptosis rate and lowered cell viability compared with doxorubicin treatment alone. Moreover, western blotting analysis demonstrated that sodium valproate increased connexin 43 (Cx43) protein expression in Hs578T BC cells [120]. Cx43 is a prominent gap junction protein within the normal human breast tissue. Cx43 plays a tumor-suppressive role in primary tumors (Table 4) [111].

Poly (ADP-ribose) polymerase 1 (PARP1) and cyclin-dependent kinase (CDK) inhibitors. PARP1 inhibitors are newly developed anticancer active agents which target cells with defects in the homologous recombination (HR) pathway. Newly developed PARP1 inhibitor AZD2461 and VPA can effectively reduce the growth of MCF-7 BC cells with no fundamental DNA repair defect. VPA and AZD2461 applied together decreased cell viability of MCF-7 cells, where IC_50_ values for VPA and AZD2461 were 4.89 mM and 42.83 µM, respectively after 48 h of treatment with active agents. Also, the trypan blue exclusion assay results showed a time- and dose-dependent decrease in cell viability in luminal BC cells after treatment with both compounds. Unfortunately, combination analysis showed a mild antagonism between VPA and AZD2461 while γ-H2AX levels were found not to be significantly increased in MCF-7 cells treated with VPA and AZD2461 together compared to each compound alone [112]. However, it has been reported that HDAC (VPA) and cyclin-dependent kinase (CDK) inhibitors (PD-033299) show synergistic interaction in BC cells and 3D cultures from pleural effusions of patients (Table 4) [113].

## 6. Valproic Acid Derivatives and Drug Carriers

VPA derivatives are promising antiproliferative agents targeting the HDAC8. Unfortunately, most of these compounds are poorly soluble in water. Therefore, G4 PAMAM, four generations of polyamidoamine dendrimers, were used to improve VPA derivatives’ water solubility. It has been demonstrated that G4 PAMAM dendrimers are capable of transporting weakly water-soluble aryl-VPA derivate compounds to increase their cytotoxicity against BC cell lines. VPA/CF-G4 PAMAM dendrimer complex shows anti-proliferative activity against MCF-7 and 3T3-L1, as well as MDA-MB-231 BC cells in the micro- and millimolar concentrations, respectively. Molecular docking and molecular dynamics simulations as well as HPLC-UV/VIS, MALDI-TOF, 1H NMR, and atomic force microscopy were used to evaluate the affinity of VPA, and its derivatives on G4 PAMAM, and then to establish the formation of the drug-G4 PAMAM complexes. HPLC UV/VIS experiments demonstrated an increase in the drug water solubility which was proportional to the G4 PAMAM amount [121]. Thus, chemical modification of VPA derivatives together with carrier development could provide a new treatment concept in the future.

## 7. Clinical Trials

Several HDIs are now being tested in BC patients in different clinical trials ranging from early phase I to randomized phase III either as single agents or in combination with approved cytostatic agents [122,123,124,125,126,127]. Despite the proven activity and high effectiveness of the use of some HDIs in hematologic malignancies [42,43,44,45], the single-agent activity seems more limited in solid tumors. Somehow, results from certain clinical trials are promising, especially those that employed VPA in combination with other chemotherapeutic drugs. Combination of VPA and epirubicin or FEC100 (5-fluorouracil, epirubicin, and cyclophosphamide), an approved regimen for BC patients, were determined [128,129]. The I phase of the study enrolled 44 patients with different solid tumors to determine the safety, toxicity, and maximum-tolerated dose of a sequence-specific combination of VPA and epirubicin. Patients were treated with increasing doses of VPA (days 1–3) followed by epirubicin (day 3) in 3-week cycles. The maximum-tolerated and recommended doses for II phase were determined (VPA 140 mg/kg/d for 48 h followed by epirubicin 100 mg/m^2^). Interestingly, sustained plasma concentrations of VPA exceeding those required for in vitro synergy were achieved with acceptable toxicity. Moreover, anticancer activity of VPA and epirubicin was observed in patients with anthracycline-resistant tumors [128]. In the II phase dose expansion enrolled 15 patients with locally advanced (IIIC) or metastatic (IV) BC (14 evaluable for response). Patients in the dose-expansion group were treated with a 120 mg/kg/day VPA loading dose followed by 60 mg/kg given every 12 h for 5 doses followed by FEC100. At dose-expansion, 9 of 14 (64%) evaluable patients had an objective response. In the trial, it has been demonstrated that a combination of VPA and FEC100 has an acceptable toxicity profile and antitumor efficacy [129]. In addition to VPA, its derivatives have been tested in clinical trials. The biological and clinical efficacy as well as the safety of magnesium valproate and hydralazine (methyltransferase inhibitor) in combination with doxorubicin cyclophosphamide in the neoadjuvant treatment of locally advanced BC, were determined. BC patients were treated with 182 mg or 83 mg of hydralazine for rapid- or slow-acetylators, respectively; and 30 mg/kg of magnesium valproate, starting from the day-7 until chemotherapy ended, the latter consisting of four cycles of doxorubicin 60 mg/m^2^ and cyclophosphamide 600 mg/m^2^ every 21 days. Needle biopsies were taken from primary tumors at diagnosis and day 8 of treatment with valproate and hydralazine. Regarding the safety of cytotoxic chemotherapy-associated magnesium valproate and hydralazine, this treatment was well-tolerated. Drowsiness in the majority of patients was the toxicity that could be attributed to the experimental therapy with valproate, however, it was not interfering with patient functioning in daily living. Interestingly, magnesium valproate and hydralazine in combination up- and down-regulated 89 and 1091 genes at least 3-fold, respectively. The results of this study demonstrate that therapy with valproate and hydralazine is safe, that it achieves the molecular changes expected from the use of HDI and demethylating agents, and appear to increase the efficacy of conventional cytostatic drugs [130]. M.D. Anderson Cancer Center is currently recruiting participants for the phase I clinical trial to determine the side effects and the maximum tolerated doses (MTDs) and dose-limiting toxicities (DLTs) of bevacizumab and temsirolimus alone or in combination with VPA or cetuximab in patients with advanced or metastatic malignancy, including BC (III and IV stages). In the clinical trial patients receive temsirolimus intravenously (i.v.) over 30–60 min on days 1, 8, 15, and 22, bevacizumab i.v. over 30–90 min on days 1 and 15, and VPA orally (p.o.) daily on days 1–7 and 15–21. The purpose of the study is the preliminary assessment of the anti-tumor efficacy of each combination, assessment of the pharmacokinetics, pharmacodynamic markers of target inhibition, and correlates of response [131].

## 8. Discussion

BC is one of the leading causes of cancer-related death among women worldwide. A significant challenge in treating BC is the limited array of therapeutic options and the rapid development of resistance against currently used agents, especially in TNBC, the most aggressive subtype of BC [97]. The idea of treating BC patients with active agents able to re-establish expression of tumor suppressor genes silenced by epigenetic mechanisms is currently being tested [132]. Up-regulated HDAC activity is associated with a closed chromatin assembly and subsequent gene repression, forming a characteristic feature of malignantly transformed cells [133,134]. Histone acetylation prevents chromatin condensation as well as makes promoters and other control elements of chromatin more accessible to different transcription factors, and thus seems to be the most important mechanism in HDIs anticancer action [135]. HDIs as the epigenetic modifiers have pleiotropic effects on many biological processes such as cancer cell growth arrest, proliferation, differentiation, angiogenesis, invasion, metastasis or immunogenicity [135]. VPA is a clinically available HDI that notably increases apoptosis, induces cell cycle arrest, and abolishes drug resistance in BC cells in vitro as well as decreases tumor growth and metastases in animal models [135]. However, divergent data on the effects of VPA on different signaling pathways, including the EMT process or miRNA pathways limit the use of this compound as a single agent in the therapy of BC, although VPA is currently still prescribed worldwide as a well-tolerated, relatively safe and effective anticonvulsant and mood stabilizer. Even though other HDIs have demonstrated more promising antitumor effects, VPA was investigated for anti-cancer activity based on its low toxicity profile and availability. It has been shown excellent tolerability of VPA within the serum range of 50–100 μg/mL based on experience from its use as an antiepileptic agent [136]. VPA was rapidly absorbed after oral administration, with peak serum levels occurring approximately 1–4 h after a single oral dose [135]. Due to the fact, that VPA has been used in clinical practice in nontoxic therapeutic concentrations in many seizure types and syndromes, and remains a mainstay for treatment of epilepsy of all age groups except for infants, as well as mania in bipolar disorders, migraine prophylaxys, neuropathic pain and schizophrenia, for more than four decades, its pharmacokinetic profile, side effects and toxicity are thus well documented [135]. Moreover, therapy with VPA is widespread, relatively cheap, and available. Since VPA, as a psychoneurological drug, crossing the blood-brain barrier, it could also effectively eliminate metastatic BC cells in the brain of patients. There is currently no effective therapy for treating metastatic TNBC in the brain. The diagnosis of an oncological disease may cause a serious psychological crisis, and the use of VPA in the treatment of BC could simultaneously reduce the symptoms of neuropsychiatric diseases. VPA is generally well tolerated by patients. However, neurological side effects such as dizziness, sedation, and tremor as well as mild gastrointestinal toxicities usually occur early during treatment. Fatal hepatotoxicity is very rare and mainly occurs in children aged less than 2 years who are treated with multiple drugs [137]. VPA is also a known human teratogen and its prescription during pregnancy (especially in the first trimester) may cause multiple birth defects that are overall designated as fetal valproate syndrome. The major congenital abnormalities are neural tube defects, facial dysmorphism, growth retardation, delay in postnatal cognitive development, and autism [135]. Despite the side effects of VPA, especially in pregnant women, clinical trials are still ongoing for its potential application in the treatment of several types of cancers, including solid and non-solid tumors [138]. While VPA administered alone demonstrated an anti-cancer effect in the pre-clinical setting, little improvement was observed with VPA in monotherapy in the clinical setting. These findings suggest that VPA needs to be combined with hormonal therapy agents or traditional chemotherapy agents in the BC setting in the future. The promising pre-clinical data suggest that VPA can be repurposed as an adjunctive agent in combination with many cytotoxic, hormonal, and immunotherapeutic agents for the treatment of BC [97]. The effect of VPA in monotherapy on induction of apoptosis, inhibition of the cell cycle, EMT or miRNA pathways do not differ significantly between the histological subtypes of BC. However, it has been demonstrated that HER2-overexpressed BC cells are more sensitive to VPA than HER2-negative. It is known that the anti-proliferative mechanism of VPA in BC cells is related to their HER2-expression status. Therefore, VPA may synergize with drugs used in the therapy of HER2-overexpressed BC, like anti-HER2 monoclonal antibodies or anthracycline and taxane-based neoadjuvant chemotherapy. Interestingly, a different therapeutic effect was observed in the VPA-combined therapy depending on the type of BC. It has been demonstrated that the anticancer effect of VPA in combination with other active agents, is highly cell-type specific. Additivity or additivity with a tendency towards synergism was demonstrated between VPA and CDDP in luminal BC cells, while antagonism was evidenced in TNBC cells between the same drug combination. Interestingly, changes in Notch1 activity in MDA-MB-231 TNBC cells caused additive interaction. Therefore, the therapy with VPA and CDDP can be a promising regimen in patients with the most aggressive type of BC-TNBC with increased Notch1 activity. Synergistic type of pharmacological interaction was also demonstrated between VPA and capecitabine or PD-033299 (CDK inhibitor) in luminal, HER2-overexpressed, and TNBC cells as well as with camptothecine in luminal BC cells. In contrast, mild antagonism was evidenced in luminal BC cells treated with VPA in combination with AZD2461 (PARP1 inhibitor). However, further investigations are warranted to evaluate the efficacy and to provide optimal treatment.

## 9. Conclusions

Despite significant progress in the therapy of BC patients, serious side effects, as well as high toxicity of standard chemotherapeutics to normal cells limit the effectiveness of the therapy. Moreover, the existence of de novo drug resistance (tumor does not respond to treatment since the beginning of therapy) or acquired resistance (response to the drug disappears over time) support the failure of standard therapies and do not bring satisfactory results [139,140,141]. Thus, a new generation of cytostatics effective in the treatment of BC is being sought, the use of which will not only reduce the doses of standard chemotherapeutics but also eliminate the phenomenon of drug resistance. Clinical trials investigating new targeted drugs as well as therapeutic combinations with their use have led to significant advances in BC therapy [7]. Epigenetic regulation of histone and non-histone proteins may be a novel approach and hold significant progress for the successful treatment of BC. HDIs are a promising class of anti-neoplastic agents that induce differentiation and apoptosis in many types of cancer cells, including breast carcinoma cells [142]. VPA is a clinically available HDI that notably inhibits migration, increases apoptosis, cell cycle arrest, and abolishes drug resistance [108] in BC cells. Unfortunately, the IC_50_ doses of VPA are relatively high compared to other HDIs in in vitro studies [59]. Moreover, divergent data on the effects of VPA on different signaling pathways, including the EMT process or miRNA pathways limit the use of this compound in monotherapy of BC. Combined therapy with the use of VPA and standard cytostatic drugs to reduce the doses of VPA and limit the adverse effects caused by standard chemotherapeutics seems to be a promising strategy in the future. Unfortunately, data on the VPA activity in combination with other anti-cancer drugs from the in vivo models is still not sufficient. Therefore, results obtained from in vitro studies should be thoroughly validated in in vivo models. Results from in vivo experiments might offer a rationale for clinical studies of a new combined therapy, to improve the clinical outcome of patients with BC.

## Figures and Tables

**Table 1:** Classification of histone deacetylases (HDACs) [8,41].

Class of HDAC	HDAC	Zinc/Nicotinamide (NAD)-Dependent
I	HDAC1, 2, 3, 8	zinc-dependent
II	IIa: HDAC4, 5, 7, 9 IIb: HDAC6, 10	zinc-dependent
III	SIRT1-SIRT7	NAD-dependent
IV	HDAC 11	zinc-dependent

**Table 2:** Classes of histone deacetylase inhibitors (HDIs) (CTCL-cutaneous T-cell lymphoma, FDA-Food and Drug Administration, PTCL-peripheral T-cell lymphoma) [10].

Class of HDIs	HDI	Abrreviation	FDA Approval for Cancer Treatment
Hydroxamic acids	Tichostatin A	TSA	Approved for CTCL treatment Approved for PTCLs treatment Approved for multiple myeloma treatment
	Vorinostat	SAHA	
	Belinostat	PXD-101	
	Panobinostat	LBH-589	
	Resminostat	4SC-201	
Short chain fatty acids	Sodium butyrate	NaB	
	Phenylbutyrate	PBA	
	Valproic acid	VPA	
Cyclic peptides	Apicidin	CAS183506-66-3	Approved for PTCLs treatment
	Romidepsin	FK228	
Benzamides	Mocetinostat	MGCD103	
	Entinostat	MS-275	
	Domatinostat	4SC-202	

**Table 3:** Mechanism of action of valproic acid (VPA) in in vitro breast cancer (BC) pre-clinical setting (BC-breast cancer, EMT-epithelial-mesenchymal transition, ER-estrogen receptor, HER-2-human epidermal growth factor receptor 2).

Cellular Process	Sub-Type of BC	Cell Line	Mechanism of Action	References
Apoptosis	Luminal	MCF7	↑apoptosis (↑p21, ↑Bak, ↑Bax/Bcl-2 ratio, ↓Bcl-2 proteins expression, ↓telomerase activity)	[60]
		MCF7 tem cells	↑apoptosis (↑M30 protein expression, ↑caspase 3 and 7 activation, ↑nuclear pycnosis)	[61]
	HER-2-overexpressed	SKBR3	↑apoptosis (↑cleaves caspase 3, ↑Hsp70 acetyaltion)	[62]
	TNBC	MDA-MB-231	↑apoptosis	[57]
Cell cycle	Luminal	MCF7	cell cycle arrest in sub-G1 phase	[60]
		ZR-75-1	cell cycle arrest in G1 phase	[60]
	HER-2-overexpressed	SKBR3	↑p21WAF1 protein expression	[62]
	TNBC	MDA-MB-231	cell cycle arrest in G0/G1 phase	[57]
Migration	Luminal	MCF7	↑migration	[63]
		MCF7 T47D	↓migration	[64]
	TNBC	MDA-MB-231	↓migration (↑*nm23H1* gene expression)	[65]
		MDA-MB-231 MDA-MB-468	↓migration	[64]
		MDA-MB-231	↑migration	[57,63]
EMT	Luminal	MCF7	↑EMT (↑*Snail*, ↑*Zeb-2* genes expression)	[63]
		MCF7 T47D	↓EMT (↑*E-cadherin* gene and protein expresssion)	[64]
	TNBC	MDA-MB-231	↑EMT (↑*Snail*, ↑*Zeb-2* genes expression)	[63]
			↑EMT (↑*Snail*, ↓*E-cadherin*, ↓*GKS3β* genes expression)	[57]
		MDA-MB-468	↑EMT (↑*N-cadherin* gene and protein expression)	[64]
miRNA	Luminal	MCF7	↑miR-34a, ↑miR-520h expression	[56]
	TNBC	MDA-MB-231	↑miR-34a, ↑miR-520h expression	[56]
ER receptor status	TNBC	MDA-MB-231	↑*ERα*, ↑*FoxA1* genes and proteins expression	[66]
Metabolic pathways	Luminal	MCF7	↑furfural expression, alteration in alanine, taurine and hypotaurine metabolism	[67]

↑—increase, ↓—decrease.

**Table 4:** Mechanism of action of valproic acid (VPA) and other anti-cancer drugs in combination in in vitro and in vivo breast cancer (BC) pre-clinical setting (CDDP-cisplatin, CDK-cyclin-dependent kinase, DXR-doxorubicin, 5-FU-5-fluorouracil, HER2-human epidermal growth factor receptor 2, N/A-not analyzed, PARP-poly (ADP-ribose) polymerase 1, TNBC-triple-negative breast cancer).

Drug-Drug Combination	BC-Subtype	Cell Line	Mechanism of Action	Type of Pramacological Interaction	References
VPA+CDDP	Luminal	MCF7	↑apoptosis, ↓cell viability, cell cycle arrest	additivity	[59]
		T47D		additivity with tendency towards synergism	[59]
	TNBC	MDA-MB-231	↑apoptosis, ↓cell viability, cell cycle arrest	antagonism	[59]
		MDA-MB-231 with decreased and increased Notch1 activity	↓cell viability	additivity	[58]
VPA+5-FU	TNBC	MDA-MB-468	sensitization of BC cells insensitive to 5-FU	N/A	[108]
VPA+capecitabine	Luminal, HER2-overexpressed, TNBC	MCF7, SKBR3, MDA-MB-231, MDA-MB-468	↓proliferation, ↑apoptosis, ↑thymidine phosphorylase gene and protein expression	synergism, additivity	[109]
VPA+camptothecin	Luminal	MCF7	↑apoptosis (↓BcL-xl protein espression)	synergism (more than additivity)	[110]
VPA+DXR	TNBC	Hs578T	↓viability, ↑cytotoxicity, ↑apoptosis, ↑Cx43 protein expression	N/A	[111]
VPA+AZD2461 (PARP1 inhibitor)	Luminal	MCF7	↓viability	mild antagonism	[112]
VPA+PD-033299 (CDK inhibitor)	Luminal, HER2-overexpressed, TNBC	Panel of BC cells (MCF7, T47D, BT474, MDA-MB-361, SKBR3, HCC1143, HCC38, HCC1806, BT483, BT549, MDA-MB-435, MDA-MB-453,) and 3D cultures from pleural effusion of patients	↑apoptosis, cell cycle arrest, overexpression of CDKN1C gene	synergism	[113]

↑—increase, ↓—decrease.
